# Hydronéphrose géante bilatérale sur syndrome de jonction pyelo-ureterale

**DOI:** 10.11604/pamj.2016.23.82.8765

**Published:** 2016-03-11

**Authors:** Anouar El Ghazoui, Othmane Yddoussalah

**Affiliations:** 1 Service d'Urologie B, CHU Ibn Sina, Rabat, Maroc

**Keywords:** Hydronéphrose, bilatérale, pyelo-uretrale, Hydronephrosis, bilateral, pyelo-ureteral

## Image en médecine

Monsieur Z.A âge de 35 ans, sans antécédents particuliers. Il avait consulté pour des douleurs lombaires bilatérales. Cliniquement il était apyrétique, BU négative, l'examen mettait en évidence une volumineuse masse occupant la région lombaire droite. Le bilan biologique avait objectivé une créatininémie à 18 mg/L, et un ECBU stérile. Sur le plan radiologique, l’échographie abdominale mettait en évidencedeux volumineuses masses rénales hydriques et cloisonnées faisant évoquer des reins multikystiques ou une hydronéphrose géante bilatérale. L'Uro scanner retrouvait une hydronéphrose géante bilatérale laminant le parenchyme rénal. Une néphrostomie bilatérale avait été réalisée et avait permis d’évacuer environ 5 litres d'urines stériles. La pyélographie antérograde était en faveur d'un syndrome de jonction bilatéral confirmer par la scintigraphie dynamique. Enfin le patient fut perdu de vue par la suite, quelques mois plus tard il revient avec une insuffisance rénale terminale; Un suivi néphrologique avait été préconisé. Nous présentons cette observation afin de montrer l intérêt du diagnostic précoce et du suivi du syndrome de jonction pyelo ureterale.

**Figure 1:**
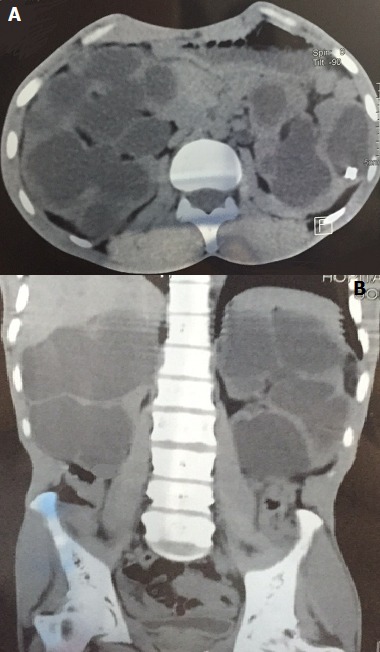
A) hydronéphrose bilatérale laminant le parenchyme rénal; B) volumineuse hydronéphrose bilatérale

